# Relations between Prosociality and Psychological Maladjustment in Chinese Elementary and Secondary School Students: Mediating Roles of Peer Preference and Self-Perceived Social Competence

**DOI:** 10.3390/bs13070547

**Published:** 2023-06-30

**Authors:** Mingxin Li, Guomin Jin, Tongyan Ren, Aersheng Haidabieke, Lingjun Chen, Xuechen Ding

**Affiliations:** 1 Department of Psychology, Shanghai Normal University, Shanghai 200234, China; 2 School of Psychology and Cognitive Science, East China Normal University, Shanghai 200062, China; 3 School of Education, Shanghai Jiao Tong University, Shanghai 200240, China; 4 Laboratory for Educational Big Data and Policymaking, Shanghai Academy of Educational Sciences, Shanghai 200032, China; 5 The Research Base of Online Education for Shanghai Middle and Primary Schools, Shanghai 200234, China

**Keywords:** prosocial behavior, psychological maladjustment, peer preference, self-perceived social competence

## Abstract

Despite empirical findings that prosociality can prevent elementary and secondary school students from developing psychological maladjustment, little is known about the underlying mechanisms. The goal of the present study was to examine the mediating effects of peer preference and self-perceived social competence on the associations between prosociality and psychological maladjustment (i.e., depressive symptoms and loneliness). Participants were 951 students (Mage = 11 years, 442 girls) in Grades 3~7 from Shanghai, China. They completed peer nominations of prosociality and peer preference and self-report measures of self-perceived social competence, depressive symptoms, and loneliness. Multiple mediation analyses revealed that: (a) both peer preference and self-perceived social competence mediated the relations between prosociality and psychological maladjustment, and (b) a serial indirect pathway (i.e., prosociality → peer preference → self-perceived social competence → psychological maladjustment) emerged when controlling for age group and gender. These findings point to potential targets in the prevention and intervention of Chinese students’ internalization of problems.

## 1. Introduction

Prosociality refers to one’s tendency to act in a manner that benefits others, and it manifests in many ways, such as caring, sharing, and helping [1]. As a major aspect of social competence, prosociality is critical to child and adolescent normative development [2]. Research has shown that elementary and secondary school students who exhibit more prosociality often report fewer internalizing symptoms, such as depressive symptoms and loneliness [3,4,5]. These studies suggest that prosociality may serve as a protective factor against becoming psychologically maladjusted. Despite consistent evidence linking prosociality to better psychological functioning, the underlying mechanisms that help to account for these links remain under-investigated [6]. Drawing from contextual-developmental perspectives [7] and informed by prior research findings [8,9], this study explores the mediating effects of peer preference and self-perceived social competence in the relations between prosociality and psychological maladjustment among a large sample of Chinese elementary and secondary school students.

### 1.1. Prosociality and Psychological Maladjustment

Engagement in prosocial behavior necessitates good emotion regulation and social–cognitive skills [2]. For example, being prosocial requires the ability to regulate one’s negative feelings when observing another’s distress [10,11]. Thus, the self-regulation abilities that prosocial students display may help them be less prone to emotional problems. A growing body of literature has examined the association between prosociality and adolescent psychological maladjustment [12,13,14], such as depression and loneliness. For example, longitudinal studies show that prosociality caused a decrease in depressive symptoms in elementary and secondary school students [3,15,16]. A recent meta-analysis also supports that prosocial behavior is negatively related to internalizing problems including depression, although the effect is small [6]. Similar to depression, students with higher prosociality tend to experience lower levels of loneliness [5,17]. Taken together, this body of research has stressed that prosocial behavior can work as a protective factor for psychological well-being. However, how prosociality relates to adolescent psychological maladjustment remains relatively unclear. Understanding the underlying mechanisms of these relations will help to understand the key variables related to prosociality that may closely affect students’ psychological well-being.

### 1.2. Peer Preference and Self-Perceived Social Competence as Potential Mediators

In addition to direct relations, prosociality is likely to be related to decreased depressive symptoms and loneliness indirectly through peer preference and self-perceived social competence. According to the contextual-developmental perspective [7], cultural values provide a basis for social evaluations of children’s behaviors, which may in turn shape children’s developmental outcomes. In Chinese society where interpersonal harmony is emphasized, prosocial behavior is highly encouraged and regarded as a behavioral virtue that contributes to effective group functioning [18]. Accordingly, students with high prosociality are likely to receive positive feedback from their peers, as shown in higher peer preference (i.e., likeability) [3,7,19]. Being well-liked and preferred by peers may provide students with more opportunities to receive instrumental help and emotional support from peers, which ultimately contribute to their fewer internalizing problems, such as depressive symptoms and loneliness [5,20]. The mediating effect of peer preference on the relation between prosociality and psychological maladjustment has been investigated once. In this study, researchers found that students with high levels of prosociality were more preferred and well-liked by their peers, which in turn reduced their depressive symptoms [3].

Aside from peer preference, self-perceived social competence may be the other potential mediator that can explain the relations between prosociality and psychological maladjustment. Self-perceived social competence refers to one’s subjective perceptions of competence or adequacy in the social domain of functioning [21]. Children’s social behaviors have long been linked to their self-perceived competence [22,23]. Prosocial students may regard their sense of self, especially their social selves, in a more favorable light. This is because taking prosocial actions may enable individuals to feel valued and needed by others, which bolsters feelings about the social self [24]. Research has shown that prosociality is linked to higher levels of general self-worth and social worth [24,25]. Cognitive theories of internalizing disorders posit that individuals with adaptive self-schemas are less vulnerable to developing internalizing problems [26,27]. The positive self-perception is thus thought to protect elementary and secondary school students from developing psychological problems [28,29].

Peer preference and self-perceived social competence may mediate the relations between prosociality and psychological maladjustment in a serial manner. The competency-based model suggests that peer experiences, such as peer preference, may impact self-perceptions of one’s ability to function in the social domain (i.e., self-perceived social competence) [26,28,30,31]. As discussed above, prosocial adolescents are usually accepted and well-liked by peers [3,7], and they may integrate positive feedback from peers into their sense of social self, which leads them to perceive that they are socially accepted and competent. This high level of self-perceived social competence, in turn, could mitigate the risk of experiencing depression and loneliness. Consistent with this theorizing, self-perceived social competence has been found to mediate the link between peer rejection and children’s internalizing problems [32]. In this study, the authors found that socially rejected children had poorer self-perceived social competence, which in turn heightened the risk for internalizing problems. Therefore, incorporating the contextual-developmental perspective, cognitive theories of internalizing disorders, the competency-based model, and the empirical evidence reviewed above, we proposed that students with high prosociality would be more preferred and accepted by their peers; subsequently, high levels of peer preference would relate to increased self-perceived social competence, which in turn reduced their experiences of depression and loneliness.

### 1.3. The Present Study

**Figure 1:**
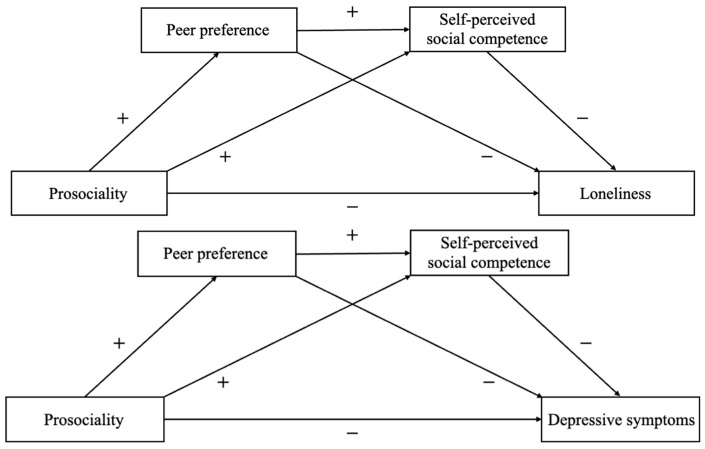
Hypothesized conceptual model.

Although previous studies found that prosociality is related to decreased depressive symptoms and loneliness among elementary and secondary school students [3,4,5], few examined the mechanisms that account for the relations. Therefore, the present study aimed to examine whether peer preference and self-perceived social competence act as mediators of the relations (see Figure 1). Grounded on the relevant theories and empirical studies reviewed above, in the present study, we propose the following hypotheses: (a) prosociality will be directly and negatively related to loneliness and depressive symptoms but positively related to peer preference and self-perceived social competence, while peer preference and self-perceived social competence related to loneliness and depressive symptoms negatively. (b) Prosociality will be linked with psychological maladjustment through its negative associations with peer preference and self-perceived social competence in a serial way. That is, prosociality would be related to greater peer preference, which in turn would be related to higher levels of self-perceived social competence. Higher self-perceived social competence would then be related to decreased depressive symptoms and loneliness (see Figure 1).

## 2. Method

### 2.1. Participants

Participants in this study were 951 students (*M*_age_ = 11 years, 442 girls, range = 8.75 years~13.34 years) in Grades 3~7 from a public elementary school and a public secondary school in the urban area of Shanghai. Both schools are typical elementary and secondary schools in urban areas of China, and almost all the students from these two schools have successfully graduated. The sample included 181 third graders (18.7%), 240 fourth graders (24.8%), 169 fifth graders (17.5%), 180 sixth graders (18.6%), and 181 seventh graders (18.7%). All participants in this sample were of the majority Han nationality.

### 2.2. Procedure

Students completed peer nominations and self-report measures that were group-administered during class time on a school day. The administration of the measures was carried out by trained researchers (i.e., graduate students). All students in the schools were invited to participate with no criteria for exclusion. Extensive explanations of the procedure and measures were provided during data collection. All subjects gave their informed consent for inclusion before they participated in the study. The study was conducted in accordance with the Declaration of Helsinki, and the protocol was approved by the Ethics Committee of Shanghai Normal University (No. 2023026). Recruitment and data collection were all completed in September 2021.

### 2.3. Measures

#### 2.3.1. Prosociality

Prosociality was measured using a peer-nomination measure adapted from the Revised Class Play [33]. Consistent with the procedure outlined by Masten [34], participants were asked to nominate up to three classmates who best fit each of the three descriptors assessing aspects of prosocial behavior (e.g., “Helps others when they need it”, “Is polite to others”). Both same-gender and cross-gender nominations were allowed, and self-nominations were not allowed. Nominations each child received from all classmates for each item were totaled and standardized within the class to form an index of prosociality. The measure was used and shown to be reliable and valid in other studies with Chinese children [35]. The internal reliability (Cronbach’s alphas) of the measure is 0.89 in this study.

#### 2.3.2. Depressive Symptoms

Participants’ depressive symptoms were assessed using a 14-item measure of the Children’s Depression Inventory (CDI) [36]. Each item consists of three alternative responses (e.g., “I feel like crying every day”, “I feel like crying most days”, and “I feel like crying once in a while”). Children were asked to choose one that best describes them in the past two weeks. Following the procedure outlined by Kovacs [36], the average score of depressive symptoms was computed, with higher scores indicative of more depressive symptoms. The measure has been demonstrated to be reliable and valid in Chinese children [3,37,38]. In this study, the internal consistency coefficient of the scale was 0.87.

#### 2.3.3. Loneliness

Loneliness was assessed by a self-report measure, adapted from Asher [39]. The 16 items assess the individual’s experiences with loneliness, such as feeling isolated, having few close relations, or feeling left out. The average score of loneliness was computed, with higher scores indicative of greater feelings of loneliness. The measure has been demonstrated to be reliable and valid in Chinese children [40,41,42]. The internal consistency coefficient of the scale was 0.92 in this study.

#### 2.3.4. Peer Preference

Participants were asked to nominate up to three classmates with whom they most liked to be around (i.e., positive nominations) and three classmates with whom they least liked to be around (i.e., negative nominations). Nominations received from all classmates were totaled and standardized within classrooms to account for different sizes of classes. Peer preference was calculated by subtracting negative nomination scores from positive nomination scores [43]. The procedure has been used in Chinese children [40,44,45].

#### 2.3.5. Self-Perceived Social Competence

Students’ self-perceived social competence was assessed using the social competence subscale of the Self-Perception Profile for Children (SPPC) [46]. The subscale consists of six items. Each item consists of two opposite descriptions (e.g., “Some kids know how to become popular” BUT “Other kids do not know how to become popular”). Children were asked to choose the description that best fits and then indicate whether the description is somewhat true or very true for them. Thus, each item is scored on a four-point scale with a higher score reflecting a higher self-perceived social competence. The measure has been demonstrated to be reliable and valid in Chinese children [47,48]. The internal consistency coefficient of the scale in this study was 0.83.

### 2.4. Analytic Plan

Data were analyzed using SPSS 25.0 and SPSS PROCESS macro 4.0 software [49]. Firstly, descriptive statistics and Pearson correlations were conducted for the main study variables. We then used the PROCESS macro to test the hypothesized multiple mediation models. In PROCESS, model six was applied to examine the mediating effect of peer preference and social self-perception on the associations between prosociality and psychological maladjustment. Mediation analyses were conducted using the 5000 bootstraps sampling method to generate 95% bias-corrected confidence intervals (CI) for all the indexes. When zero was not included in the 95% CI, the effects were considered statistically significant. Age group and gender were included in the models as covariates.

## 3. Results

### 3.1. Descriptive Statistics

Means and standard deviations for and intercorrelations among study variables are presented in Table 1. The results showed that prosociality was positively correlated with self-perceived social competence and peer preference but negatively correlated with loneliness and depressive symptoms, whereas self-perceived social competence and peer preference were negatively correlated with loneliness and depressive symptoms.

A multivariate analysis of variance (MANOVA) was conducted to examine the overall effects of gender (boys, girls), age group (elementary school, secondary school), and their interactions on study variables. Results indicated that there were significant main effects of gender, Wilks’ λ = 0.94, *F* (5, 893) = 10.01, *p* < 0.001, and age group, Wilks’ λ = 0.94, *F* (5, 893) = 10.01, *p* < 0.001. Follow-up univariate analysis revealed that compared to boys, girls had higher scores on peer preference, *F* (1, 891) = 15.13, *p* < 0.001, *η*^2^ = 0.01, and prosociality, *F* (1, 891) = 58.32, *p* < 0.001, *η*^2^ = 0.05. Secondary school students had higher levels of depression than students in elementary school, *F* (1, 891) = 25.21, *p* < 0.001, *η*^2^ = 0.05. There was also a significant interaction between gender and age, Wilks’ λ = 0.97, *F* (5, 893) = 4.29, *p* < 0.001. Specifically, boys had higher level of depression and loneliness than girls in elementary school, whereas girls had higher score of both depression and loneliness in secondary school (depressive symptoms: (*F* (1, 897) = 5.79, *p* < 0.05, *η*^2^ = 0.01); loneliness: (*F* (1, 897) = 15.12, *p* < 0.001, *η*^2^ = 0.02). For self-perceived social competence, girls had higher self-perceived social competence than boys in elementary school, *(F* (1, 897) = 6.82, *p* < 0.05, *η*^2^ = 0.01), but boys had higher self-perceived social competence than girls in secondary school. On the other hand, girls had higher scores on prosocial behavior and peer preference in elementary school (prosociality: *F* (1, 897) = 9.12, *p* < 0.05, *η*^2^ = 0.01; peer preference: (*F* (1, 897) = 7.81, *p* < 0.05, *η*^2^ = 0.01), but there was no significant difference in secondary school.

### 3.2. Testing Multiple Mediation Models

**Figure 2:**
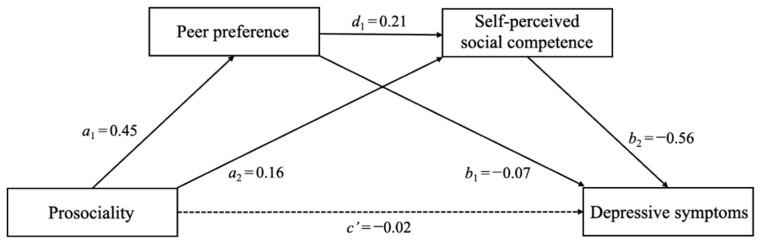
The multiple mediation role of peer preference and self-perceived social competence in the relation between prosociality and depressive symptoms. Note: Values reflect standardized coefficients. Age and gender were controlled for as covariates.

**Figure 3:**
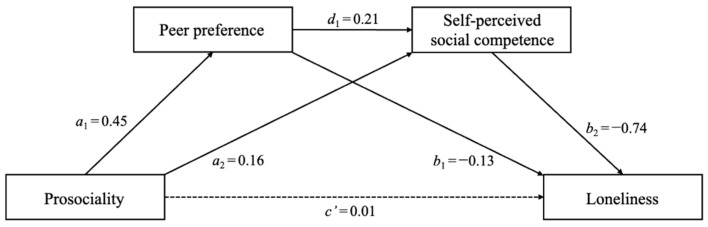
The multiple mediation role of peer preference and self-perceived social competence in the relation between prosociality and loneliness. Note: Values reflect standardized coefficients. Age and gender were controlled for as covariates.

Model six of PROCESS was used to test our hypothesized multiple mediation models, with separate models evaluated for each dependent variable. The results of our models are shown in Figure 2 and Figure 3 and Table 2. In the model with depressive symptoms as the dependent variable (Figure 2), prosociality was positively related to peer preference (a_1_ = 0.45, *p* < 0.001), which, in turn, was negatively related to depressive symptoms (b_1_ = −0.07, *p* = 0.025). The indirect effect of prosociality on depressive symptoms through peer preference was significant (a_1_b_1_ = −0.03, 95% CI [−0.06, −0.01]), thus demonstrating that peer preference served as a mediator. In addition, prosociality was positively related to self-perceived social competence (a_2_ = 0.16, *p* < 0.001), which, in turn, was negatively related to depressive symptoms (b_2_ = −0.56, *p* < 0.001). The indirect effect of prosociality on depressive symptoms through self-perceived social competence was significant (a_2_b_2_ = −0.09, 95% CI [−0.13, −0.05]), thus demonstrating that self-perceived social competence served as a mediator. Moreover, peer preference was positively related to self-perceived social competence (d_1_ = 0.21, *p* < 0.001). The serial mediating effect of prosociality on depressive symptoms through peer preference and self-perceived social competence was also significant (a_1_d_1_b_2_ = −0.05, 95% CI [−0.07, −0.04]). 

In the model with loneliness as the dependent variable (Figure 3), prosociality was positively related to peer preference (a_1_ = 0.45, *p* < 0.001), which, in turn, was negatively related to loneliness (b_1_ = −0.13, *p* < 0.001). The indirect effect of prosociality on loneliness through peer preference was significant (a_1_b_1_ = −0.06, 95% CI [−0.09, −0.04]), thus demonstrating that peer preference served as a mediator. In addition, prosociality was positively related to self-perceived social competence (a_2_ = 0.16, *p* < 0.001), which, in turn, was negatively related to loneliness (b_2_ = −0.74, *p* < 0.001). The indirect effect of prosociality on loneliness through self-perceived social competence was significant (a_2_b_2_ = −0.12, 95% CI [−0.17, −0.07]), thus demonstrating that self-perceived social competence served as a mediator. Moreover, the serial mediating effect of prosociality on loneliness through peer preference and self-perceived social competence was also significant (a_1_d_1_b_2_ = −0.07, 95% CI [−0.09, −0.05]).

## 4. Discussion

Previous research has shown that prosociality is critical to child and adolescent social functioning [2]. Elementary and secondary school students with high levels of prosociality often report lower levels of internalizing symptoms, such as depressive symptoms and loneliness, which suggest that prosociality may serve as a protective factor for developing psychological maladjustment [3,4,5]. However, the mechanisms that might help to explain the association between prosociality and psychological maladjustment are not well understood. Especially considering the function of peer preference and self-perceived social competence during preadolescence and early adolescence. Accordingly, in the present study, we evaluated a complex conceptual model to examine the mediating effects of peer preference and self-perceived social competence on the associations between prosociality and psychological maladjustment. Multiple mediation analyses indicate that the association between prosociality and psychological maladjustment is mediated by both peer preference and self-perceived social competence. Additionally, a serial indirect pathway was observed when controlling for age group and gender.

### 4.1. The Mediation Role of Peer Preference and Self-Perceived Social Competence

In this study, it was found that prosocial behavior may prevent elementary and secondary school students from psychological symptoms. According to the results of this study, peer preference mediates the relations between prosociality and psychological maladjustment. It is consistent with previous findings that showed the reciprocal association between prosocial behaviors and peer relations, namely, positive social behaviors promote good peer relations and vice versa [50,51]. Moving beyond that, peer difficulties, such as low social preference, have been shown to affect children’s symptoms of depression from kindergarten [52]. This effect may even last through adolescence [53]. The link from prosocial behavior to peer interaction and then to psychological adjustment can be explained by the contextual-developmental perspective [7]. Cultural values offer a framework for social assessments of children’s behaviors, which can subsequently shape their developmental trajectories. In Chinese culture, where promoting interpersonal harmony is emphasized, prosocial behavior is encouraged and esteemed as a moral virtue that facilitates the smooth functioning of the peer network [18]. Consistently, studies had reported that students with prosocial behaviors (such as politeness, helping others, leadership, etc.) generally had supportive peers and were favored by teachers. [54,55]. These positive interaction experiences enable them to meet their own social, psychological, and instrumental needs and their emotional experience of decrease in depression and loneliness accordingly. This finding provided new evidence for the relations between prosocial behavior and psychological symptoms through peer preference, which is in line with the perspective of contextual-developmental. In Chinese culture, helping others is a sign of personality sublimation, and students who interact with each other under this value will be preferred by their peers and thus reducing their depressive symptoms and loneliness.

In the present study, self-perceived social competence is another potential mediator that can explain the relations between prosociality and psychological maladjustment. Self-perceived social competence can be defined as the degree to which people’s judgments of how they are seen by others are correct [56]. Children’s social behaviors have long been linked to their self-concept, and studies have found a significant correlation between self-concept and cooperative behavior [17,18,57]. Prosocial behavior enables individuals to retain their sense of self, especially their social selves, in a more favorable light because they may feel valued and needed by others, bolstering feelings about the social self eventually [24]. On the other hand, according to sociometer theory, perceptions of peer approval are regarded as essential for assessments of one’s worth as a person. Further, adolescents start to evaluate themselves on a variety of life domains, including their social roles and integration in peer groups [29,58,59]. Consistent with prior findings, this study provided evidence that prosocial behavior can be used as a protective factor to allow individuals to experience less psychological maladjustment. The high level of prosociality is positively connected with self-perceived social competence, which mirrored the results that prosociality is linked to higher levels of general self-worth and social worth [24,25], and the positive self-perceived social competence, in turn, may protect elementary and secondary school students from developing psychological problems [28].

### 4.2. The Serial Multiple Mediation Model

This study further found that peer preference and self-perceived social competence play a serial mediation role between prosociality and psychological maladjustment. It provides evidence of a positive connection between peer preference and self-perceived social competence, which is partly consistent with previous research; adolescents who report being rejected by their peers have higher levels of depression and loneliness and lower levels of self-esteem and perceived self-competence [14,30]. Humanism emphasizes the individual and social potential and agency of human beings. Humanists believe that loneliness is an individual’s subjective feeling about the number of friends and quality of friendship as well as the evaluations of their basic social skills. Students feel lonely when the quantity and quality of their social networks are lower than expected [60]. Hymel and colleagues have explained the relations between loneliness and social status from the perspective of social cognition. They believe that children’s loneliness and their actual social status among peers is the individual’s perceived level of interpersonal relations mediated by social cognition [61]. Dodge devised a model of social information processing, which stresses the cognitive steps in evaluating social situations. Those students who have defects or deviations in social information processing will encounter difficulties in interacting with their peers and be negatively evaluated by their peers and vice versa [62]. Different social statuses will cause elementary and secondary school students to know the world and themselves in different ways. Rejected students may adopt self-defensive attributions, and they will also adopt withdrawal methods when solving problems and interacting with peers, resulting in the deviated cognition of social networks and themselves [61]. Similar to the previous studies, this analysis found that students with high social self-perception have significantly lower loneliness than students with low self-perceived social competence [54]. Group feedback that students with different social statuses received can also affect children’s cognitive and behavioral responses, thereby causing changes in children’s self-perception, including social self-perception [61]. As mentioned above, under the cultural orientation of harmony in China, prosocial students are well-liked. These students are more likely to develop positive self-perception, including social self-perception, which can reduce adjustment difficulties consequently.

## 5. Conclusions

The limitations and suggestions of this study are as follows. First, although the proposed mediation models are grounded in strong theoretical frameworks and empirical studies, the possibility of reciprocal relations between some study variables cannot be ruled out due to the cross-sectional nature of our data. For example, the relations between prosociality and depressive symptoms may be bidirectional [3], and effect of prosociality, peer preference, and self-perceived social competence on psychological maladjustment may be reciprocal. Relatedly, there may be biases when mediation effects are examined using cross-sectional data [62]. To address these limitations, it is recommended that future research adopt a multiple-wave longitudinal design, which will allow for the testing of mediation effects as well as the examination of directional and transactional processes over time [63]. Second, data were derived from self-reports, and the social desirability effect may affect the accuracy of the results. In the future, prosocial behavior and psychological maladjustment together with their associations can be studied through interviews and other methods. Third, children in their preadolescence and early adolescence phase are in an important stage of individual development, and their prosocial behavior is easily affected by other factors in their daily life, such as their development of the theory of mind and popularity goals [64,65]. Therefore, in the future, these variables could be included as an endorsement of boundary conditions in this model to further explore the impact of other factors on prosocial behavior and the psychological mechanism. Fourth, this study had a limited age range from middle childhood to early adolescence, and the long-term developmental differences could not be explained. In the future, studies can use an extended age group from early childhood to adulthood.

In conclusion, our study tested a serial mediation model of peer preference and self-perceived social competence as mediators between prosocial behavior and psychological maladjustment among Chinese elementary and secondary students. The results suggested that prosociality may be related to greater levels of peer preference, which in turn may be associated with higher levels of self-perceived social competence. Higher self-perceived social competence would then be related to decreased psychological maladjustment (i.e., depressive symptoms and loneliness) among students of elementary and secondary school in Chinese culture. The mechanisms of these relations may inform prevention and early intervention programs for internalizing problems through the strength of prosociality among elementary and secondary school students. Role play and social cognition training about prosocial behaviors can shape students’ perceptions, especially self-perceived social competence, correctly. Additionally, subjective evaluation (self-perceived social competence) of peer interaction and objective assessment from peers (nominations from their social group) are important characteristics of peer relations to include in research investigating peer relations in adolescence [62]. Therefore, the current study assessed peer relations from both subjective and objective perspectives when examining their links between prosocial behavior and psychological symptoms, which extends the field of the effect of peer interaction on psychological maladjustment.

## Figures and Tables

**Table 1:** Means, standard deviations, and correlations among all variables (N = 951).

Variables	*M*	*SD*	Correlations				
			1	2	3	4	5
1. prosociality	0.00	1.00	1				
2. loneliness	1.92	0.73	−0.23 **	1			
3. depression	1.41	0.35	−0.17 **	0.64 **	1		
4. SPSC	2.96	0.69	0.25 **	−0.77 **	−0.59 **	1	
5. PP	0.00	1.59	0.46 **	−0.032 **	−0.22 **	0.28 **	1

Notes: SPSC = Self-Perceived Social Competence; PP = Peer Preference; M = mean value; SD = standard deviation. ** *p* < 0.01.

**Table 2:** Total, direct, and indirect effects of prosociality (X) on depressive symptoms and loneliness (Y) through peer preference (M1) and self-perceived social competence (M2).

Dependent Variable	Effect	Estimate	SE	95% CI	
				Lower	Upper
Depressive symptoms					
	Total effect	−0.19	0.03	−0.2593	−0.1284
	Direct effect	−0.02	0.03	−0.0757	0.0445
	Total indirect effect	−0.18	0.02	−0.2221	−0.1323
	Indirect effect (X→M1→Y)	−0.03	0.02	−0.0608	−0.0009
	Indirect effect (X→M2→Y)	−0.09	0.02	−0.1292	−0.0531
	Indirect effect (X→M1→M2→Y)	−0.05	0.01	−0.0742	−0.0357
Loneliness					
	Total effect	−0.24	0.03	−0.3047	−0.1720
	Direct effect	0.01	0.02	−0.0332	0.0629
	Total indirect effect	−0.25	0.03	−0.3005	−0.2001
	Indirect effect (X→M1→Y)	−0.06	0.01	−0.0827	−0.0322
	Indirect effect (X→M2→Y)	−0.12	0.03	−0.1714	−0.0733
	Indirect effect (X→M1→M2→Y)	−0.07	0.01	−0.0957	−0.0464

## Data Availability

The datasets generated during and/or analyzed during the current study are available from the corresponding author on reasonable request.
